# Commentary: GSK-3 Inhibition as a Therapeutic Approach Against SARs CoV2: Dual Benefit of Inhibiting Viral Replication While Potentiating the Immune Response

**DOI:** 10.3389/fimmu.2020.595289

**Published:** 2020-10-19

**Authors:** Andre De Souza, Fabio A. Tavora, Devalingam Mahalingam, Pamela N. Munster, Howard P. Safran, Wafik S. El-Deiry, Benedito A. Carneiro

**Affiliations:** ^1^Division of Hematology/Oncology, Lifespan Cancer Institute, Warren Alpert Medical School of Brown University, Providence, RI, United States; ^2^Argos Laboratory, Fortaleza, Brazil; ^3^Division of Hematology/Oncology, Northwestern University, Chicago, IL, United States; ^4^Department of Medicine (Hematology/Oncology), Helen Diller Family Comprehensive Cancer Center, University of California, San Francisco, San Francisco, CA, United States; ^5^Department of Pathology and Laboratory Medicine, Alpert Medical School of Brown University, Providence, RI, United States

**Keywords:** glycogen synthase kinase-3 (GSK3), glycogen synthase kinase-3 (GSK-3) inhibitor, COVID, COVID-19, 9-ING-41, GSK-3b inhibitor, GSK-3 inhibitor, SARS-CoV21

We read with interest the insightful manuscript by Christopher E. Rudd highlighting the potential of Glycogen Synthase Kinase-3 (GSK-3) inhibitors for the treatment of SARS-CoV2 (1). The manuscript describes the GSK-3-mediated phosphorylation of key serine residues in SARS-CoV2 nucleocapsid proteins essential for viral replication. These results coupled with preclinical evidence demonstrating the role of GSK-3 in the modulation of innate and adaptive immune responses support the author's hypothesis that GSK-3 inhibitors could be investigated as a potential treatment for COVID-19 infection. GSK-3 small-molecule inhibitors and GSK-3 siRNA reduced PD-1 expression, increased CD8 + T cell function, and enhanced viral clearance in models of herpes (MHV-68) and lymphocytic choriomeningitis (LCMV-C13) viral infections (2). The increased T cell function induced by GSK-3 inhibition resulted in anti-tumor activity comparable with the effects observed with anti-PD-1 monoclonal antibodies in animal models of metastatic melanoma and lymphoma (3). The therapeutic relevance of GSK-3 inhibition is also suggested by studies utilizing animal models of hemorrhagic shock showing that GSK-3β inhibition dampens liver and renal dysfunction by upregulation of anti-inflammatory IL-10 and down-regulation of IL-12p40 and IL-6, a cytokine implicated in the cytokine release syndrome observed in patients with severe SARS-CoV2 (4). GSK-3β inhibition also attenuates the systemic inflammatory response (SIR) in models of sepsis and ischaemia/reperfusion injury by modulating NF-kB-induced inflammatory response (5–8). These findings have potential implication to SIR and the frequent disseminated intravascular vascular coagulopathy observed during the infection with SARS-CoV2. GSK-3β inhibition also decreased mRNA expression of IL-1β, IL-6, and inducible NO synthase (iNOS) in a model of lipopolysaccharide (LPS) mediated inflammation (9).

Dr. Rudd discusses the therapeutic potential of specific GSK-3 inhibitors (i.e., SB216763, tideglusib) and proposes lithium as a GSK-3β inhibitor to consider for clinical trials, an available oral drug with known toxicity profile. However, lithium has a narrow therapeutic index and is significant less potent than other small-molecule GSK-3β inhibitors in clinical development (10, 11). For instance, the potent and selective ATP-competitive GSK-3β inhibitor 9-ING-41 has advanced to the clinic with preliminary results from ongoing clinical trials involving patients with advanced malignancies demonstrating an excellent safety profile devoid of myelosuppressive and immunosuppressive effects. We have recently presented the initial data from an ongoing phase I/II clinical trial of 9-ING-41 administered as monotherapy or combined with several chemotherapy regimens at the annual meeting of the American Society of Clinical Oncology (NCT03678883). The study has enrolled over 200 patients with advanced malignancies and completed cohorts with monotherapy and combination with various standard chemotherapy regimens without attributable Grade 3 or 4 serious adverse events (12). Of particular relevance to a potential COVID-19 study, 9-ING-41 is not associated with myelosuppression of any degree and there has been no evidence of increased infection or any opportunistic/unusual infections even in patients with extensive prior cytotoxic therapy. A complete response was documented in a patient with BRAF V600E metastatic melanoma previously treated with dabrafenib/trametinib and nivolumab, including resolution of brain metastases. Evidence of durable responses and prolonged treatment duration among patients with pancreatic cancer receiving 9-ING-41 plus gemcitabine/nab-paclitaxel and endometrial cancer receiving 9-ING-41 plus carboplatin/paclitaxel has supported the recent activation of a phase 2 study investigating the former regimen in the front-line treatment of metastatic pancreatic cancer with other Phase 2 studies in preparation. In addition to its anti-cancer activity, 9-ING-41 also reduced pulmonary fibrosis and improved pulmonary function in models of TGF-β and bleomycin pulmonary fibrosis (13, 14). This anti-fibrotic activity supported the clinical development and ongoing clinical trial of 9-ING-41 in patients with advanced myelofibrosis (NCT04218071). Considering the likely high global burden of fibrotic lung disease following the pandemic, GSK-3β inhibition with 9-ING-41 could also have a favorable impact in reducing the lung sequelae from COVID-19 infection. Recent autopsy studies from China, Italy and US have demonstrated marked fibrotic lung disease in patients who suffered from the COVID-19 (15–17). Results from *in vitro* and xenograft models of ovarian cancer also demonstrated that 9-ING-41 was more active than lithium and other ATP-competitive inhibitors such as SB216763 (the compound utilized in the experiments showing the enhancement of T cell function) (10). While ongoing studies are evaluating the immune modulatory effects of 9-ING-41 in T cell function, experiments in prostate cancer cell lines showed that 9-ING-41 regulated the expression of PD-L1 (18). Based on 9-ING-41's established safety profile and pre-clinical potent anti-fibrotic activity aligned with the robust preclinical rationale for GSK-3β blockade outlined elegantly by Dr. Rudd, we believe that this novel GSK-3β inhibitor merits urgent consideration as an investigational therapy for patients with clinically significant COVID-19 infection.

## Author Contributions

All authors listed have made a substantial, direct and intellectual contribution to the work, and approved it for publication.

## Conflict of Interest

The authors declare that the research was conducted in the absence of any commercial or financial relationships that could be construed as a potential conflict of interest.
